# Robot-Embodied Neuronal Networks as an Interactive Model of Learning

**DOI:** 10.2174/1874205X01711010039

**Published:** 2017-09-30

**Authors:** Abraham M Shultz, Sangmook Lee, Mary Guaraldi, Thomas B. Shea, Holly A. Yanco

**Affiliations:** 1 Robotics Laboratory, Department of Computer Science, USA; 2 Laboratory for Neuroscience, Department of Biological Sciences University of Massachusetts Lowell, Lowell, MA 01854, USA

**Keywords:** Multi-electrode array, Neuronal network, Cortical neuronal culture, Learning, Plasticity, Sensory input, Cybernetics, Manus robot arm

## Abstract

**Background and Objective:**

The reductionist approach of neuronal cell culture has been useful for analyses of synaptic signaling. Murine cortical neurons in culture spontaneously form an *ex vivo* network capable of transmitting complex signals, and have been useful for analyses of several fundamental aspects of neuronal development hitherto difficult to clarify *in situ*. However, these networks lack the ability to receive and respond to sensory input from the environment as do neurons *in vivo*. Establishment of these networks in culture chambers containing multi-electrode arrays allows recording of synaptic activity as well as stimulation.

**Method:**

This article describes the embodiment of *ex vivo* neuronal networks neurons in a closed-loop cybernetic system, consisting of digitized video signals as sensory input and a robot arm as motor output.

**Results:**

In this system, the neuronal network essentially functions as a simple central nervous system. This embodied network displays the ability to track a target in a naturalistic environment. These findings underscore that *ex vivo* neuronal networks can respond to sensory input and direct motor output.

**Conclusion:**

These analyses may contribute to optimization of neuronal-computer interfaces for perceptive and locomotive prosthetic applications. *Ex vivo* networks display critical alterations in signal patterns following treatment with subcytotoxic concentrations of amyloid-beta. Future studies including comparison of tracking accuracy of embodied networks prepared from mice harboring key mutations with those from normal mice, accompanied with exposure to Abeta and/or other neurotoxins, may provide a useful model system for monitoring subtle impairment of neuronal function as well as normal and abnormal development.

## 1 INTRODUCTION

The advent of culture dishes within which is embedded an array of electrodes has allowed interfacing of cultured neurons with computer software for recording and stimulation. This robust approach has been utilized to gain major advances in understanding of synaptogenesis and neuronal signaling [1-6], as well as gaining insight into factors that promote these processes and underlie or accelerate neurodegeneration [7-9]. These Multi-Electrode Arrays (MEA) and the appropriate software have demonstrated that cultured neurons spontaneously undergo synaptogenesis and establish a functional network capable of transmitting information across multiple neurons. Over the course of approximately 1 month, the spontaneous signal patterns generated by these *ex vivo* neuronal networks undergo developmental conversion from individual “spikes” to complex bursts that consist of multiple signals [3-6, 10-15]. This conversion recapitulates the delayed establishment of inhibitory neuronal activity observed *in situ* [16-21].

External stimulation hastens the establishment of a signaling pattern characteristic of mature cultures [2, 3, 12, 22-24], confirming that *ex vivo* networks are capable of responding to external stimulation. Our studies in which networks were subjected to multiple stimulation regimens using a digitized synaptic signal further demonstrated that *ex vivo* networks altered their signaling patterns in a manner analogous to long-term potentiation *in situ* [6].

A more robust and “real-world” approach towards activity and responsiveness of *ex vivo* neuronal networks has been to interface them with robotics [25, 26]. Herein, we provided *ex vivo* neuronal networks with sensory input using a digital video camera, and converting resultant network signaling to impulses capable of operating a robotic arm. The resultant “embodied” neuronal network therefore represents a simple central nervous system that processes incoming sensory input and generates corresponding motor output.

## 2 MATERIALS and METHODS

### 2.1 Generation of *Ex Vivo* Neuronal Networks

Dissociated cortical neurons from day 18 C57BL/6 mouse embryos were plated in B27-supplemented Neurobasal medium (Invitrogen, Carlsbad, CA) on poly-D-lysine/fibronectin-coated “MEA petri dishes” (Multichannel Systems, Reutlingen, Germany) containing 60 Titanium Nitride electrodes in an 8 by 8 grid arrangement; detailed information on generation of networks and of the MEAs themselves has been published previously [3-6, 27]. Sacrifice of pregnant females was carried out under procedures approved by our Institutional Animal Care and Use Committee.

### 2.2 Recording of Network Activity

Signaling was recorded using a MEA-1060-INV amplifier over 30 second intervals and collected *via* a DT9814 acquisition system and a software program (“Raptor”) developed in our laboratory [3, 4] (available at https://github.com/ mtgjbird/Raptor). Raptor requires the LabView (National Instruments) run-time engine 2013 SP1 or later http://www.ni.com/download/labview-run-time-engine-2013-sp1/4540/en/. Other appropriate software is available [1, 28]. Networks were stimulated using a 1sec digitized synaptic signal 1mV in amplitude as in our prior studies [3, 4, 27]. Output was graphed and analyzed in Excel.

After culturing for 1 month to allow maturation of networks (confirmed by development of spontaneous signal streams rich in complex bursts [27], networks were segmented into two equivalent halves by slicing across the petri dish with a scalpel. This segmentation was not essential, but served to generate two independent “hemispheres.” Separation was confirmed by phase-contrast microscopy Fig. (**1**) and by confinement of responding signals following stimulation within the half containing the electrode to which the exogenous signal was delivered (not shown). Networks were utilized immediately after segmentation, and therefore there was not sufficient time for regeneration of connections across the slice.

### 2.3 Interfacing of Ex Vivo Neuronal Networks with Video Input and Robotic Output

The complete software package, developed in our laboratory, and used for real-time interfacing our ex vivo neuronal networks with digital video input and robotic output is available at https://github.com/ab3nd/ NeuronRobotInterface.

### 2.4 Conversion of Video Signals and Stimulation of Neuronal Networks

This was performed by the ROS module, “img_slicer,” developed in our laboratory (available at https://github.com /ab3nd/NeuronRobotInterface/ tree/master/catkin_ws/src/img_slicer/src).

We utilized a solid red cup as a target object within a defined visual field. Notably, an object of any shape or hue could be utilized. Video was recorded with a tripod-mounted RGB digital camera. The resulting video was converted into HSV (Hue, Saturation, Value), and separated into hue, saturation, and value planes, which generated an accompanying image for each plane. This results in three images, one for each plane. The resultant hue image was subjected to thresholding to convert all pixels of the desired hue (red, in the example utilized herein) were converted to white, and all other pixels were converted to black (*e.g.,* Fig. (**2**).

To monitor movement of the target, the overall image field was separated into five equivalent vertical strips. The total white pixels in the leftmost 3 strips was then compared with the total in the rightmost 3 strips. If the sum of white pixels in the leftmost 3 strips was 500 more than the sum in the rightmost 3 strips, stimulation (*via* a digitized synaptic signal) [27] was delivered to the right of the network, in order to foster increased activity in the right portion of the network and vice versa. Accordingly, each difference of 500 pixels was translated into one synaptic signal. Delivery of synaptic signals to one half of the segmented network increased the activity of that segment Fig. (**2**); delivery of sufficient synaptic signals to increase the activity of that half of the network by a sufficient level prompted delivery of signals to the robot arm as described below.

### 2.5 Conversion of Neuronal Network Activity to Motion Commands for the Manus Robotic Arm

This was performed by the ROS module, “act_vector,” developed in our laboratory (available at: https://github.com/ ab3nd/NeuronRobotInterface/tree/master/ catkin_ws/src/activation_vector/src).

Each of the 60 channels of the MEA was recorded for 3sec intervals at a rate of 1000 samples/sec. The mean and standard deviation of signaling on each channel was calculated, with one signal defined a >3x the standard deviation of that channel.

A 60-element list (one for each channel) of activation values was initialized at zero. When a set of samples arrive, all the activation values A of each channel n were reduced according to the equation *A_n_*(*t_i_*) = *A_n_*(*t_i_*−1)*e*^−^*^β^*^(^*^ti^*^−^*^ti^*^−1)^ as described by Hales *et al.* 2010. For each channel where a signal is detected, the activation value was incremented by 1. Every 0.2 sec, the list of activation vectors was compared to the predefined “left” (L) and “right” (R) activation vectors by taking the 60 dimensional Euclidian distance between the current activation vector and the L and R vectors; these are fixed vectors reflecting increased activity on the L or R sides of the network. If the distance between the L or R activation vector approximates one of the fixed vectors (indicating that network activity was stronger on either the L or R side), a corresponding motion command was sent to the Manus robot arm, otherwise a stop command was sent to the Manus robot arm.

## 3 RESULTS

**Fig. (1):**
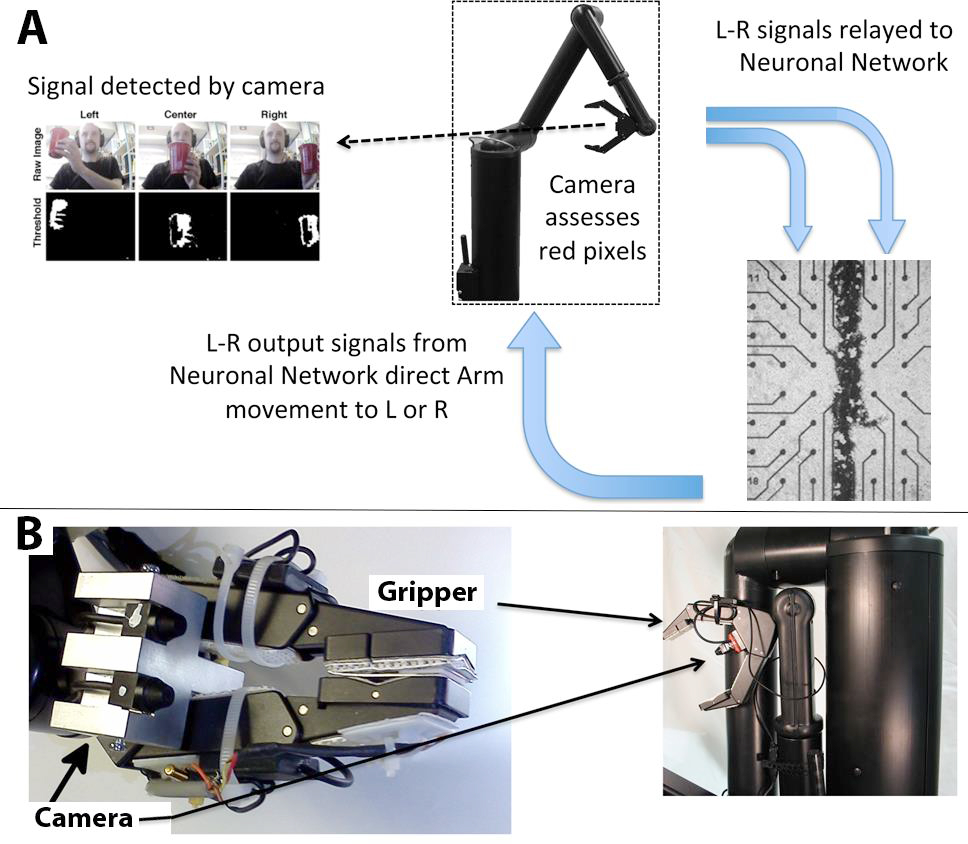
Diagram of the embodied neuronal network.

A diagram of information flow through the embodied neuronal network is presented in (Fig. **1**).

Panel A describes basic information flow through the components of this system as described in Methods. The camera detects the position of the target, and transfers signals to the appropriate half of the segmented network. The stimulated network transmits motion commands to the arm, and the arm repositions in the direction of the target. This sequence repeats until the arm is situated directly in front of the target (if the target is stationary) or continues to track the target (if the target is moving). A portion of the segmented neuronal network is presented; black dots are the electrodes.

Panel B depicts the digital camera mounted above the grasping hand of the Manus robot arm.

Representative results of camera input and resultant network activity are presented in Fig. (**2**); as can be seen, visualization of the target increased network activity according to its localization.

**Fig. (3):**
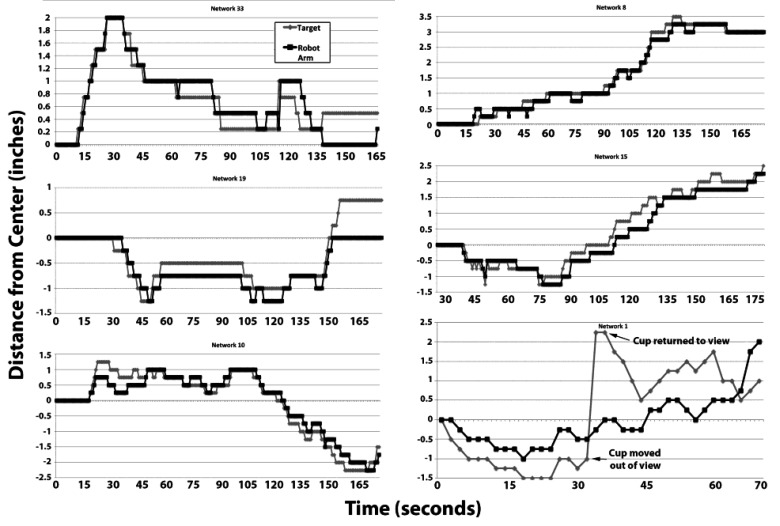
**Representative tracking of the target by the robot arm.** Mov files of tracking were opened to an identical size, and a ruler was mounted below the on-screen image, with 0 representing the initial localization of the robot arm. Migration of the target and arm were quantified over the indicated time period. Migration was quantified using distance increments of 0.25”; + values indicate migration to the right, - values indicate migration to the left.

Tracking of the target by multiple representative networks is presented in Fig. (**3**). Visual inspection of these sequences indicates that the robot arm followed the target. Notably, in one of these sequences (network 1), the target was purposely moved out of view of the camera, at which point the robot arm returned to 0 (the midpoint) due to absence of differential signals between the network halves. Tracking resumed when the target was moved back into view (Fig. **3**).

**Fig. (4):**
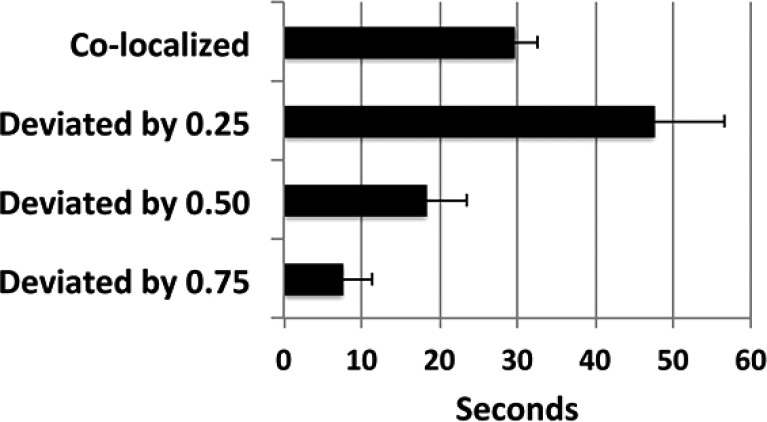
**Average accuracy of tracking**. The period of time during which the robot co-localized within the same 0.25” area as the target, or deviated from the target by various distances, is indicated.

Analyses of tracking in these examples demonstrated that the robot arm tracked the target with considerable accuracy; the arm remained within the smallest unit measured (<0.25”) for 68 ± 13% of the observation period (Fig. **4**).

## 4 DISCUSSION

We present herein a model with unique positioning of an *ex vivo* neuronal network to receive digital environmental input, and to provide responsive output. Since the input corresponds to sensory information, and the output corresponds to motor activity, the *ex vivo* network functions as a population of interneurons – *i.e.*, a central nervous system that processes incoming information and generates an appropriate response. This unique model can be analyzed in detail, and/or expanded upon to encompass additional functionality.

Prior studies demonstrated that these networks display alterations in signaling consistent with long-term potentiation following repetitive stimulation with the same digitized synaptic signal utilized herein [4, 6]. Embodiment of the neuronal network as described herein provides the unique opportunity for future studies of long-term potentiation by biological neuronal networks in an environmental context. Many studies have resorted to artificial networks, which provide an environment that can readily and precisely be manipulated, but retain the limitation that they are not comprised of actual neurons. Previous studies have utilized similar *ex vivo* networks to operate a small model vehicle within a closed environment and observed network firing patterns in response to physical barriers [25, 26]. The current system expands upon this “reactive” earlier embodiment of neuronal networks in our system monitored active tracking of a target versus passive response.

Setting a threshold for full red pixels allowed us to track the target against a background of mixed coloration that included an additional non-target object that contained red pixels (the human operator). Use of a solid-color background that lacks non-target objects would lessen/eliminate a requirement for thresholding, but would impose an artificial situation that is not reminiscent of real-world dynamics. With the “mixed” background utilized herein, we did not attempt to identify intermediate threshold settings, the minimum target size/shape, degree of additional non-target objects with varying degrees of red pixels, and whether or not the target could contain regions of other hues, all of which would be of interest.

It should be noted that perfect co-localization is not to be anticipated, since the robot responds to net movement of the target; *i.e.,* the target itself must move a distance of >0.25” in either direction to stimulate corresponding movement of the arm. Tracking of the arm could instead be accomplished by digital analyses of target and arm movement. However, this approach involves considerable “stuttering,” due to minor differences in pixel recording. Accordingly, we considered the most appropriate method for monitoring tracking was instead to record net movement of the arm within pre-defined increments Fig. (**4**). An additional compounding factor was that endogenous network activity continued during tracking regimens, which prevented stimulation of the robot arm exclusively by exogenously-derived stimulation.

We have conducted full-range tracking (*i.e.,* full left to full right and back again), as well as maintaining our target exclusively within the left or right regions, with similar tracking in both cases. In one instance in which we removed the target from the field of view, the robot arm returned to the center, and resumed tracking when the target was restored within the field of view (Fig. **4**, Network 1).

This is reminiscent of normal brain activity and perception, since synaptic activity persists throughout the brain, over which sensory input and resultant downstream activity is superimposed.

Similarly, optimal tracking was demonstrated by networks that displayed similar levels of spontaneous activity within the left and right domains. Since we converted pixels to our previously-recorded synaptic signal, and did not amplify them above their recorded amplitude, the responsiveness of the robot arm can be diminished should the network display uneven spontaneous activity. In such cases, net movement of the arm favors the side of the network with greater spontaneous activity.

Organotypic (slice) cultures can be established on MEAs [3]. However, networks established from dissociated neurons as used herein can be maintained for several months (and longer under optimal conditions) [25]. Networks generated herein have no architectural restrictions during axonogenesis, which therefore fosters nearest-neighbor connections. Future refinements will include the use of nanocages at plating, to provide a degree of directionality to developing networks. Additional possibilities include establishing of networks from various transgenic mouse models, as well as adding additional neurons to established networks as a model of neurogenesis.

In the particular configuration utilized herein, video input was derived from the digital camera mounted on the Manus robot arm. However, the MEA/computer interface could receive input from any digital camera. “Motor” output could vary from our use of a robot arm to any mechanical/functional activity with an electronic interface.

Future studies could involve a robot-embodied model containing two neuronal networks. The first network, representing the central nervous system, will control tracking of a target *via* the Manus arm as above. Following successful location of the target, an equivalent number of signals are received by and transmitted from each half of the network, and the arm therefore ceases movement. When no delta in signals between L and R hemispheres is detected (*e.g.* for ≥5 sec), the second network will be activated. Signal streams from the second network, representing the peripheral (motor neuron) system, will then direct the Manus arm to reach forward and retrieve the object; note that the Manus arm has existing programs to support retrieval and object placement. While this represents a simple two-stage embodiment, it parallels our real-world behavior in that humans locate an object of interest *via* sensory input and motor output (orientation of eyes, head and/or body), and only after localization do we stimulate a second set of motor nerves to initiate object retrieval. Moreover, our recognition of a target occurs over and above a “basal” continuous sensory input, which is analogous to the continuous spontaneous activity of networks during target recognition herein.

## CONCLUSION

Ultimately, more complex structures than those possible using individual neuronal networks can be developed, allowing exploration of the basic principles about the formation of higher-level neuronal structures. These analyses may contribute to optimization of neuronal-computer interfaces for perceptive and locomotive prosthetic applications. Manipulation of inhibitory neuronal activity and initial connectivity may provide insight into perturbations of balance in excitatory/inhibitory activity that promote seizures and/or perturb motor activity in epilepsy and Parkinson’s disease [4-6, 29]. In addition, the inherent potentially-interfering basal activity of an amplitude matching sensory input in our system may provide a useful system for analyses of conditions such as attention deficit hyperactivity disorder, where competing sensory and internal distractions can distract an individual from task recognition and completion.

*Ex vivo* neuronal networks as generated herein display rapid inhibition of complex signal patterns in response to sub-cytotoxic concentrations of oligomerized Abeta, and that these alterations are synergistically increased by iron and airborne nanoparticles, yet attenuated by zinc [7-9]. Comparison of tracking accuracy of embodied networks prepared from mice harboring key mutations with those from normal mice, accompanied with exposure to Abeta and/or other neurotoxins, may be particularly useful for monitoring subtle impairment of neuronal function.

## Figures and Tables

**Fig. (2):**
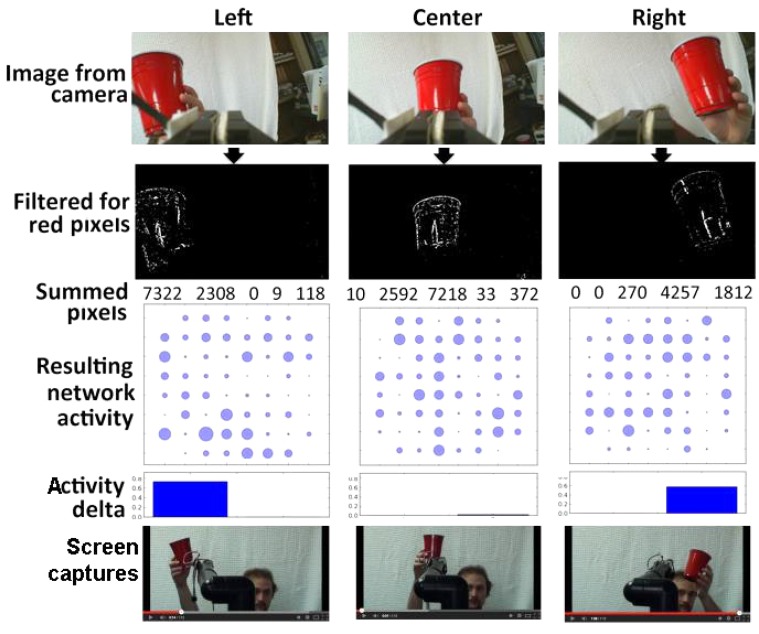
**A diagrammatic flow of information from the target to the network.** Panels present the raw image of the target (a red cup in this example), the threshold image detected by the camera, the summation of pixels across the image (“Summed pixels”), a representative image of electrode activity as a result of image input (“Resulting network activity”), and a summation of total activity, which highlights the difference in activity within the left and right portions of the networks (“Activity delta”). Also presented are representative screen captures that demonstrate the robot arm tracking the target.
